# The differences of atrial thrombus locations and variable response to anticoagulation in nonvalvular atrial fibrillation with ventricular cardiomyopathy

**DOI:** 10.1002/joa3.12422

**Published:** 2020-08-29

**Authors:** Hao Zhang, Miao Yu, Yu Xia, Xiaofeng Li, Jun Liu, Pihua Fang

**Affiliations:** ^1^ Chinese Academy of Medical Sciences and Peking Union Medical College Fuwai Hospital Beijing China; ^2^ Department of Cardiology Chongqing General Hospital University of Chinese Academy of Sciences Chongqing China

**Keywords:** anticoagulation, atrial fibrillation, atrial thrombus, location of thrombus, ventricular cardiomyopathy

## Abstract

**Objectives:**

This study aims to research the clinical features of atrial thrombi in patients with nonvalvular atrial fibrillation (AF).

**METHODS:**

This study included 191 patients of AF who had atrial thrombi. One hundred and twenty‐eight of them were assigned into nonventricular cardiomyopathy group (non‐VCM), and the remaining 63 into ventricular cardiomyopathy group (VCM). After atrial thrombi diagnosed, all patients had taken oral anticoagulant therapy. The resolution rates of thrombi within 12 months were compared between the two groups, as well as the locations of thrombi.

**Results:**

Of all 191 patients, 161 had thrombi only detected in left atrial appendage (LAA), 20 in both left atrium (LA) and LAA, six in LA only, and four in right atrium only. More patients had thrombi out of LAA in the VCM group than in the non‐VCM group (30.2% vs 8.6%, *P* < .001). After propensity score matching, the atrial thrombi were resolved faster in the non‐VCM group than in the VCM group (mean time length: 22 ± 2 weeks vs 30 ± 3 weeks, *P* = .038), and the resolution rate within 12 months was higher in the non‐VCM group than in the VCM group (88.7% vs 61.4%, Log‐rank, *P* = .038). In Cox proportional hazards model, absence of ventricular cardiomyopathy was an independent predictor for the resolution of atrial thrombus (hazard ratio: 1.76; *P* = .035).

**Conclusions:**

The patients of atrial fibrillation with ventricular cardiomyopathies have higher incidence of thrombosis in the body of left atrium or right atrium. And the resolution rate was lower in these patients.

## Graphical abstract

The most important finding is that, the ventricular cardiomyopathy is an independent risk for atrial thrombi existing in patients with atrial fibrillation.

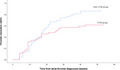

## 1 INTRODUCTION

Atrial fibrillation (AF) is a common cardiac arrhythmia, and the estimated age‐standardized prevalence of AF was 0.78% in men and 0.76% in women in a Chinese investigation.^1^ AF is associated with the development of atrial thrombus, especially in the left atrial appendage (LAA), which is considered the most common source of stroke and systemic embolisms.^2^, ^3^ However, in some patients with AF, the atrial thrombi can take place out of LAA, and the outcomes of resolution may vary from different conditions. Consequently, we performed a retrospective study to research the clinical features and variable responses to anticoagulation in AF patients complicated with cardiomyopathy and ischemic heart disease.

## 2 Methods

### 2.1 Study population and groups

Consecutive patients were retrospectively assessed from Fuwai Hospital between May 2011 and June 2018. The criteria for inclusion were: (1) Nonvalvular atrial fibrillation; (2) Age ≥18 years old; (3) First time to detect atrial thrombi in Fuwai hospital. The exclusion criteria were: (1) Valvular atrial fibrillation; (2) History of cardiac surgery. This study was approved by our institutional ethics committee. The atrial thrombi were diagnosed by at least one of the following imaging methods: transesophageal echocardiography (TEE), cardiac computed tomographic angiography, cardiovascular magnetic resonance imaging, or transthoracic echocardiography (TTE) to diagnose right atrial thrombus.

Participants in this study were assigned into two groups: ventricular cardiomyopathy group (VCM) and nonventricular cardiomyopathy group (non‐VCM). Cardiomyopathy is a myocardial disorder that the heart muscle is structurally or functionally abnormal in the absence of some secondary disorders as defined by European Society of Cardiology working group ^4^. In our study, the VCM group included patients with these cardiomyopathies and ischemic heart disease.

### 2.2 Comparison of clinical features

The baseline characteristics were compared between the two groups mentioned above, including left atrial antero‐posterior diameter (LAAPD), left ventricular end diastolic diameter (LVEDD) and left ventricular ejection fraction (LVEF) measured by echocardiography. And CHA2DS2‐VASc, CHADS2, HAS‐BLED scores were also collected as well as antithrombotic regimens before diagnosis.

### 2.3 Follow up

After atrial thrombi diagnosed, patients had taken oral anticoagulants with either vitamin K antagonist or non‐vitamin K oral anticoagulants (NOACs), which included rivaroxaban and dabigatran in this study. The patients were scheduled to outpatient clinic at 1, 3, 6, 9 and 12 months, and the same imaging method was repeated for spotting thrombi, until thrombi resolved.

### 2.4 Comparison between the VCM and NON‐VCM groups

Propensity score matching was used to compare the resolution rate of thrombi between VCM and non‐VCM groups. The propensity score was generated according to potential covariates, including gender, age, type of AF (paroxysmal or persistent), BMI, history of stroke, history of congestive heart failure, history of hypertension, history of diabetes mellitus, CHA2DS2‐VASc scores, LAAPD, LVEF, and concentration of NT‐pro‐BNP. Nearest‐neighbor matching without replacement was performed. After matching, Multivariate analysis was used to evaluate independent risk factors affecting the resolution of atrial thrombi in both matching groups. Survival curves were compared with Kaplan‐Meier method using a log‐rank test, in order to estimate the time length for thrombi resolution.

### 2.5 Statistical analysis

Statistical analyses were conducted using the SPSS version 26 (SPSS Corp., Armonk, NY, USA). Continuous variables are expressed as mean ± SD, and using the independent‐sample T test. Categorical variables are expressed as numbers or percentages, using the Chi‐square test or the Fisher's exact test. A P‐value less than 0.05 was considered statistically significant.

## 3 RESULTS

### 3.1 Clinical characteristics of participants

We had retrieved 1153 patients diagnosed with atrial fibrillation and atrial thrombus, and 962 patients were excluded because of valvular atrial fibrillation or cardiac surgery. One hundred and ninety‐one patients of AF were eligible in our study. There were 128 patients in non‐VCM group and 63 in VCM group. The patients in VCM group had larger LAAPD than in non‐VCM group (48 ± 8 mm vs 43 ± 5 mm, *P* < .001), and LVEF was lower in the VCM group than in the non‐VCM group (49% ± 15% vs 60% ± 6%, *P* < .001). More patients suffered congestive heart failure in VCM group than in non‐VCM group (60.0% vs 26.2%, *P* < .001), and the NT‐pro‐BNP concentration was higher in VCM group than in non‐VCM group (2309.1 ± 3093.8ng/mL vs 964.9 ± 1071.7ng/mL, *P* = .001). The baseline clinical characteristics and examination results of these patients are shown in Table 1.

**Table 1:** Baseline Demographic and Clinical Characteristics

Characteristics	Non‐VCM group (n = 128)	VCM group (n = 63)	*P* value
Age‐yrs	60.4 ± 10.1	58.1 ± 12.7	.171
Female‐no.(%)	37(28.9)	24(38.1)	.200
BMI (kg/m^2^)	26.3 ± 3.2	24.6 ± 3.6	.001
CHA2DS2‐VASc scores	2.7 ± 1.9	3.0 ± 1.8	.427
Has‐bled scores	1.8 ± 1.3	1.5 ± 1.1	.203
Non‐paroxysmal AF‐no. (%)^a^	84(65.6)	39(61.9)	.614
NT‐pro‐bnp (pg/ml)	964.9 ± 1071.7	2309.1 ± 3093.8	.001
laapd (mm)	43 ± 5	48 ± 8	<.001
LA < 45mm‐no.	81	24	—
LA ≥ 45mm‐no.	47	39	—
LVEDD (mm)	49 ± 4	52 ± 11	.050
LVEF (%)	60 ± 6	49 ± 15	<.001
Antithrombotic drugs‐no. (%)			
No antithrombotic drugs	31(24.2)	15(23.8)	.950
Anti‐platelet	40(31.2)	20(31.7)	.945
vka	23(18.0)	12(19.0)	.856
noacs	34(26.6)	16(25.5)	.863
Prior medical history‐no.(%)			
Congestive heart failure	35(27.3)	37(58.7)	<.001
Hypertension	78(60.9)	34(54.0)	.358
Diabetes mellitus	29(22.7)	13(20.6)	.751
Ischemic stroke	33(25.8)	14(22.2)	.591
Type of cardiomyopathy‐no. (%)			
Ischemic heart disease	—	26(41.3)	—
Dilated cardiomyopathy	—	14(22.2)	—
Hypertrophic cardiomyopathy	—	15(23.8)	—
Restrictive cardiomyopathy	—	5(7.9)	—
Arrhythmogenic right ventricular cardiomyopathy	—	2(3.2)	—
Chronic myocarditis	—	1(1.6)	—

Abbreviations: BMI denotes body mass index; NT‐pro‐BNP denotes N‐terminal pro b‐type natriuretic peptide; LAAPD denotes left atrial anterior‐posterior diameter measured by TTE; LVEDD denotes left ventricular end‐diastole diameter measured by TTE; LVEF denotes left ventricular ejection fraction measured by TTE.

^a^ The non‐paroxysmal atrial fibrillation includes persistent, long‐standing persistent and permanent atrial fibrillation in this study.

### 3.2 Distribution of thrombi site in atrium

**Figure 1:**
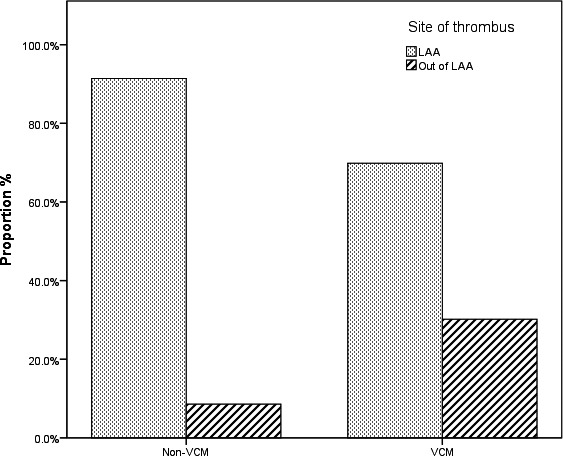
Different distribution of thrombus in ventricular cardiomyopathy group and non‐cardiomyopathy group. There is a larger proportion of thrombi located outside left atrial appendage（LAA） in VCM group than in non‐VCM group([19/63],30.2% vs [11/128],8.6%)

**Figure 2:**
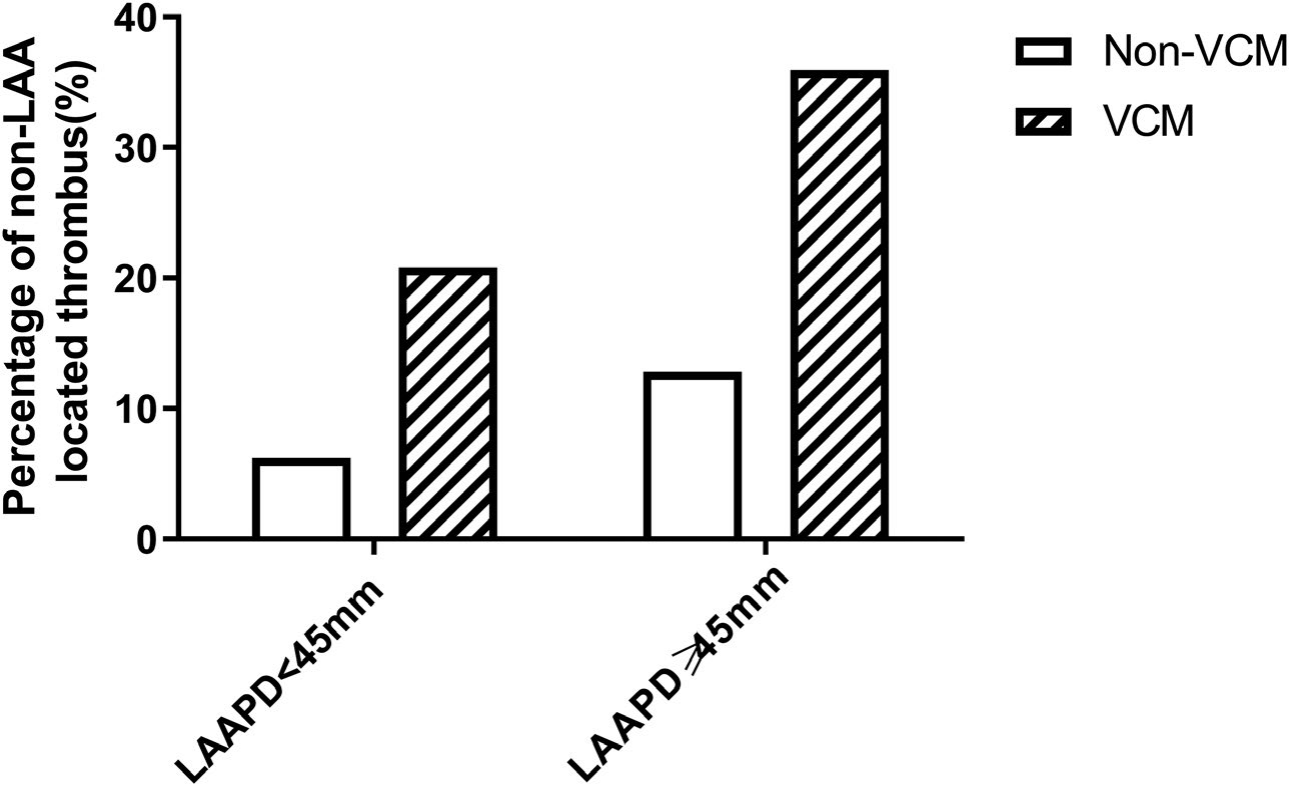
Subgroup analysis stratified by LAAPD. More patients with VCM had thrombi out of LAA than non‐VCM in the subgroup of LAAPD <45 mm ([5/24],20.8% vs [5/81],6.2%), and similar result was seen in the subgroup of LAAPD ≥45 mm ([14/39],35.9% vs [6/47],12.8%)

Of all 191 patients, 161 had thrombi only detected in left atrial appendage (LAA), 20 in both left atrium (LA) and LAA, 6 in LA only, and 4 in right atrium only. More patients had thrombi out of LAA in VCM group than in non‐VCM group (30.2% vs 8.6%, *P* < .001), and the 4 right atrial thrombi were all from VCM group. The results were shown in Figure 1. Additionally, we stratified the patients by LAAPD into two subgroups: subgroup 1 of LAAPD <45 mm, and subgroup 2 of LAAPD ≥45 mm. More patients with VCM had thrombi out of LAA than non‐VCM in subgroup 2 (35.9% vs 12.8%, *P* = .011), and similar result was seen in subgroup 1 between two groups (20.8% vs 6.2%, *P* = .047) with significance, as shown in Figure 2.

### 3.3 Anticoagulant therapy

Before the atrial thrombi detected, 55.5% of patients (106/191) did not take any oral anticoagulants, including 31.4% only taking anti‐platelet drugs (Table 1). In these 106 patients, 37.7% belonged to the high risk of stroke with CHADS2 score ≥2 points, or 70.8% with CHA2DS2‐VASc score ≥2 points. After atrial thrombi diagnosed, all patients had taken oral anticoagulant therapy. One hundred and six patients took warfarin, 64 patients took rivaroxaban, and the remaining 21 took dabigatran.

### 3.4 Follow up of thrombi resolution

After anticoagulant therapy, the median time length for thrombi resolution was 25 ± 1 weeks. The atrial thrombi was resolved faster in non‐VCM group than in VCM group (mean time length: 22 ± 1 weeks vs 32 ± 1 weeks, *P* < .001), and the resolution rate in one year was higher in non‐VCM group than in VCM group (85.9% vs 61.9%, Log‐rank, HR 2.13, 95% CI, 1.44 ~ 3.14,*P* < .001).

**Figure 3:**
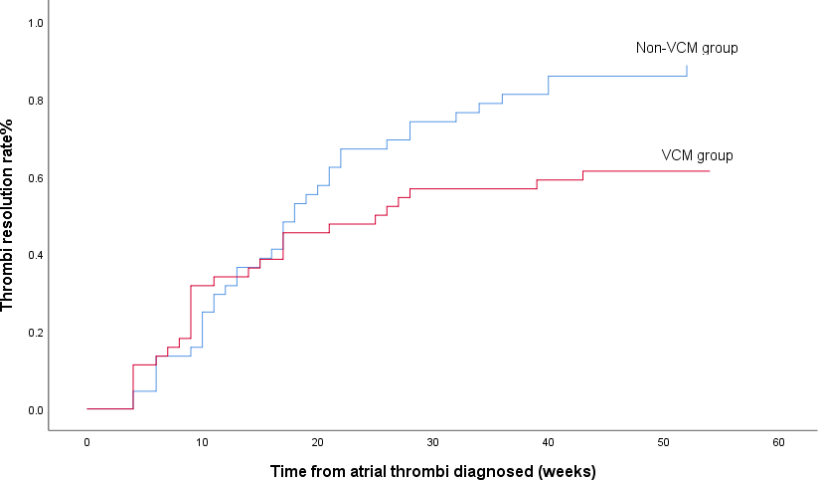
Kaplan‐Meier analysis of thrombi resolution between two PSM groups, and there were 44 patients in each group. In one‐year follow‐up, there were more thrombi resolved in non‐VCM group than in VCM group

In the propensity score‐matching analysis, 44 patients in the VCM group and 44 patients in the non‐VCM group were matched and analyzed. There was no significance in these basic clinical characteristics of 88 patients after PS matching, as shown in Table 2. In Kaplan‐Meier analysis of the two matching groups, the atrial thrombi was resolved faster in non‐VCM group than in VCM group (mean time length: 22 ± 2 weeks vs 30 ± 3 weeks, *P* = .038), and the resolution rate in one year was higher in non‐VCM group than in VCM group (88.7% vs 61.4%, Log‐rank, *P* = .038), as shown in Figure 3.

**Table 2:** Clinical Characteristics of 88 patients after PS matching

Characteristics	Non‐VCM group (n = 44)	VCM group (n = 44)	*P* value
Age‐yrs	59.4 ± 8.6	60.2 ± 12.5	.729
Female‐no.(%)	19(43.2)	19(43.2)	1.000
BMI (kg/m^2^)	25.1 ± 3.3	25.2 ± 3.5	.843
CHA2DS2‐VASc scores	2.9 ± 1.9	3.0 ± 1.9	.782
Has‐bled scores	1.6 ± 1.1	1.6 ± 1.1	.853
Non‐paroxysmal aF‐no. (%)	27(61.4)	26(59.1)	.828
NT‐pro‐bnp (pg/ml)	1359.9 ± 205.0	1606.7 ± 1721.5	.453
laapd (mm)	45 ± 5	46 ± 8	.795
LVEDD (mm)	49 ± 4	49 ± 11	.777
LVEF (%)	57 ± 7	56 ± 10	.710
Prior medical history‐no.(%)			
Congestive heart failure	18(40.9)	20(45.5)	.667
Hypertension	19(43.2)	20(45.5)	.830
Diabetes mellitus	31(70.5)	33(75.0)	.632
Ischemic stroke	35(79.5)	35(79.5)	1.000

Note

The propensity score was generated according to potential covariates, including gender, age, type of AF (paroxysmal or persistent), BMI, history of stroke, history of congestive heart failure, history of hypertension, history of diabetes mellitus, CHA2DS2‐VASc scores, LAAPD, LVEF and concentration of NT‐pro‐BNP.

In univariate analysis of these 88 patients, there were less patients with ventricular cardiomyopathy in the thrombus resolution group than in the nonresolution group (27/65 [41.5%] vs 17/23 [73.9%], 95% CI, *P* = .008). The concentration of NT‐pro‐BNP was higher in the nonresolution group than in the resolution group (1899.5 ± 1565.6 vs 1334.2 ± 1525.7 pg/mL, *P* = .133), with more females (13/23 [56.5%] vs 25/65 [38.5%], *P* = .133) and diabetes mellitus (20/23 [87.0%] vs 44/65 [67.7%], *P* = .075) patients in the nonresolution group than in the resolution group, but these were not significantly different. In the subsequent multivariate analysis, absence of ventricular cardiomyopathy (hazard ratio: 1.76; *P* = .035) was an independent predictor for atrial thrombus resolving.

## 4 DISCUSSION

Few studies had focused on the relationship between atrial thrombus and cardiomyopathy in atrial fibrillation, and the main findings from this retrospective study are as follows:

1. The atrial thrombi tend to arise out of LAA among atrial fibrillation patients with ventricular cardiomyopathy.
2. The atrial thrombi in patients with ventricular cardiomyopathy were more difficult for resolution than those without ventricular cardiomyopathy.
3. Ventricular cardiomyopathy was an independent risk for atrial thrombi existing in AF patients.

### 4.1 Difference of thrombosis locations in atrium

LAA is the most common location for thrombosis and a source of cerebral or systemic embolism in AF patients.^5^, ^6^ In patients under regular anticoagulation, the prevalence of left atrial thrombus varies from 1.5% ~ 3.0%.^7^, ^8^ In a recent meta‐analysis, the results showed that left atrial thrombus was identified in up to 9.8% of overall patients with AF, and is associated with a 3.5‐fold increased risk of stroke/systemic embolism.^2^ As shown in past studies, during AF there is a decrease in LAA contractility and function, which is prone to thrombosis.^9^, ^10^

However, in our study, 15.7% of cases had thrombi formed in LA body or right atrium, and in patients with ventricular cardiomyopathy, this proportion was up to 30%. We supposed that it may be due to the atrial remodeling in patients with ventricular cardiomyopathy.

In our study, the mean anteroposterior diameter of LA was larger in the ventricular cardiomyopathy group than in the non‐cardiomyopathy group, indicating more left atrial remodeling. Moreover in order to elucidate whether this difference was just due to the enlargement of LA, we conducted the subgroup analysis. And we found that, whether in the subgroup of LA ≥45 mm or LA <45 mm, the proportions of non‐LAA thrombi were both significantly higher in the patients with VCM than in non‐VCM. These results implied that, the cardiomyopathy may contribute to thrombi formation in the body of LA or right atrium rather than just enlargement of LA.

In some types of cardiomyopathies, such as dilated cardiomyopathy and ischemic heart disease,^11^, ^12^, ^13^ the pathologic changes (ie fibrosis) were not only manifested in the ventricular myocardium, but also in the atrium. These lesions were possibly linked to the stasis in atrium by inflammation and (or) endothelial dysfunction,^14^ which may promote thrombosis in the body of LA.

Based on the results, we inferred that, left atrial appendage closure may be unsuitable for some particular patients of AF. As in 2019 AHA/ACC/HRS guideline, left atrial appendage closure may be considered in some selected patients with AF at increased risk to prevent stroke.^15^ However, it is a controversial measure for its efficacy. From this study, we found that LAA is not the only one part for thrombosis, while some thrombi could emerge in the right atrium or the body of LA, hence the occlusion of LAA may be unable to reduce thrombosis out of LAA.

### 4.2 Anticoagulation therapy before atrial thrombus detected

In our study, over half of these patients did not take anticoagulants until the atrial thrombi detected. Nearly 25% of the patients were admitted before 2015, during which period, guidelines did not establish the status of CHA2DS2‐VASc scoring for stroke risk evaluation, and still considered aspirin for some selective patients with AF.^16^, ^17^, ^18^ In these patients without anticoagulating, over 60% were stratified into low risk with CHADS2 <2 points, which may contribute to the deficiency of anticoagulation. By revaluation through CHA2DS2‐VASc scoring, up to 70% of the patients without pre‐anticoagulation were stratified into high risk of stroke with CHA2DS2‐VASc ≥2 points. Thus, CHA2DS2‐VASc may promote anticoagulation by lifting the risk rating of stroke, thus to decrease atrial thrombosis.

### 4.3 Resolution of atrial thrombi

As guidelines recommend, VKA is the most used treatment,^15^, ^19^ and in some trials, NOACs were also proved to be effective for treating atrial thrombi in patients with AF.^20^, ^21^, ^22^ Wang et al conducted a meta‐analysis and found that, the pooled resolution rate of left atrial thrombus from 16 warfarin studies was 63.7%, and two studies in direct‐acting oral anticoagulants reported this resolution rates of 89.5% for dabigatran and 41.5% for rivaroxaban.^23^ In our study, the total resolution rate in one year was about 74% after propensity score matching, and the resolution rate in one year was higher in non‐VCM group than in VCM group. The difference of resolution rate may be related to the discrepancy of LA size and LVEF between the two groups. As a result, we conducted the univariate and multivariate analysis from the propensity score matching, and made most variates of baseline consistent. The result showed that the absence of VCM was the only independent predictor for thrombi resolution.

As discussed above, the left atrial diameter was greater in VCM group than in non‐VCM group, and the atrial contractility declined in some patients with cardiomyopathy. In addition, the patients with cardiomyopathy in our study had lower mean LVEF and higher incidence of heart failure than patients without cardiomyopathy, which could further increase dysfunction of left atrium.^24^, ^25^ All these causes led to slow blood flow in circulation, especially the left atriums, which may increase the difficulty for thrombi resolution.

### 4.4 Study limitations

This study was retrospectively conducted in a single center, and the sample size was relatively small, especially in the cardiomyopathy group. Hence, the limited size may overestimate the difference of atrial thrombus locations between the two groups. Moreover about 20% of the participants were diagnosed only by CT. As previous study indicated, CT had a poorer positive predictive value for thrombus in left atrial appendage compared with the gold standard of TEE.^26^ It may include some false‐positive thrombi into this study.

## CONFLICT OF INTEREST

This work was funded by Peking Union Medical College Youth Research Fund, except which, the authors declared that they have no conflict of interest with other people or organizations that can inappropriately influence this work.
